# Osteomyelitis variolosa, an issue inherited from the past: case report and systematic review

**DOI:** 10.1186/s13023-021-01985-0

**Published:** 2021-08-06

**Authors:** Jinshuo Tang, Pu Shao, Te Liu, Xinggui Wen, Yeliang Wang, Chenyu Wang, Yachen Peng, Hua Yao, Jianlin Zuo

**Affiliations:** 1 Department of Orthopeadics, China-Japan Union Hospital of Jilin University, 126 Xiantai Street, Changchun, 130033 Jilin China; 2 Scientific Research Center, China-Japan Union Hospital of Jilin University, Changchun, 130033 Jilin China; 3 Department of Hand Surgery, China-Japan Union Hospital of Jilin University, Changchun, 130033 Jilin China

**Keywords:** Osteomyelitis variolosa, Smallpox, Case report, Systematic review

## Abstract

**Background:**

Osteomyelitis variolosa is a self-limiting disease triggered by variola virus that cannot be prevented or repaired. Smallpox has been eradicated for 40 years, and complications that remain after smallpox has been cured have become a remarkable diagnostic challenge for contemporary physicians. In this systematic review, we searched PubMed (MEDLINE), Web of Science, and Google Scholar for cases on complications, diagnosis, and treatment for osteomyelitis variolosa between January 1980 and February 2021.

**Results:**

Ten papers and eleven finished cases, all patients from India, were included for comparison with the present case. In total, 100% of patients presented with bilateral elbow deformities, the ankle was the second most common site of lesion in 50%, and knee lesions accounted for 25% in this study. Flexion contracture, joint instability, secondary arthritis, and fracture are common complications of osteomyelitis variolosa, and most patients receive conservative treatment, while internal fixation has good results for combined fractures.

**Conclusions:**

Although osteomyelitis variolosa is not a direct threat to the safety of patients, severe skeletal deformities can have a significant impact on quality of life. With advances in surgical techniques, clinicians are offering an increasing number of treatment options for patients with osteomyelitis variolosa. However, most importantly, smallpox has basically been removed from the historical arena, and for areas where smallpox was once endemic, physicians need to deepen the understanding of this disease again.

## Background

Smallpox is an acute infectious disease that is the preserve of people alone and is caused by a poxvirus [1]. It is one of the most lethal diseases, with a mortality rate of up to 30% for the virus variant variola [2]. High-throughput shotgun sequencing data obtained from human remains indicated that variola virus was already widespread in the Viking Age in northern Europe [3]. Based on continuous improvements in vaccinology and immunology, smallpox became the world’s first infectious disease to be destroyed by humans through prophylactic administration of live vaccinia virus [4, 5]. Variola virus was declared eradicated by the World Health Organization (WHO) in 1980 and is now present in two laboratories, which were approved by the WHO [1, 6]. Uprooting smallpox is one of the outstanding achievements in the history of medicine, which has saved the lives of hundreds of millions of people. Nevertheless, we are still seeing a particular and essential issue inherited from the past in some survivors who had smallpox, that is, bone and joint deformities caused by osteomyelitis variolosa.

Osteomyelitis variolosa is a self-limiting illness that cannot be prevented or repaired [7]. During smallpox epidemics, 2–5% of children infected with smallpox exhibited osteoarticular deformities [8], which are usually symmetrical and multiloculated, most commonly in the elbows, wrists, ankles, hands, and feet and less commonly in the spine and pelvis [9]. Fortunately, however, skeletal deformities are often the net result of osteomyelitis variolosa rather than a serious threat to life safety [7]. Late skeletal manifestations of bone disease in smallpox can be detected years after the initial infection. Osseous abnormalities appear mainly in the form of tubular bones, flail joints, subluxations, dislocations, gross bony deformities, premature osteoarthritis, and joint ankyloses [9, 10]. Modern medicine, especially rapid advances in medical culture and surgical techniques, provides new options, such as arthroplasty and osteotomy [11, 12], for the treatment of patients with skeletal deformities due to osteomyelitis variolosa. However, the imaging and clinical features of these skeletal lesions vary, and it is difficult to correlate the patient’s current status with a previous history of smallpox, which makes diagnosis and treatment difficult for clinicians. In this study, we review the literature published from 1980 to 2020 and report a case of bilateral knee deformity due to osteomyelitis variolosa combined with secondary osteoarthrosis, thus providing a reference for the clinical diagnosis and treatment of such patients.

## Case report

In January 2020, a 70-year-old woman came to the China-Japan Union Hospital of Jilin University for the treatment of bilateral knee pain with limited movement that had been aggravated for approximately 1 year. She was 1.67 m tall, weighed 60.00 kilos, and with a BMI of 25.0. This patient was able to perform daily activities independently without assistance of others, but sometimes required the use of assistive devices. On physical examination, bilateral enlarged knees were seen, and the right lower extremity was 2 cm shorter than the left. A pronounced right genu varum was seen. When pressure is applied to bilateral distal femurs, proximal tibias and patella, significant unevenness of the bone surface can be felt. The right knee and left knee were passively flexed to 90° and 130°, respectively. And according to the manual muscle testing (MMT), the myodynamia of bilateral lower extremities was grade IV. In addition, this patient also had flexion contracture of the elbow joints, but it did not have a clear impact on quality of life.

**Fig. 1:**
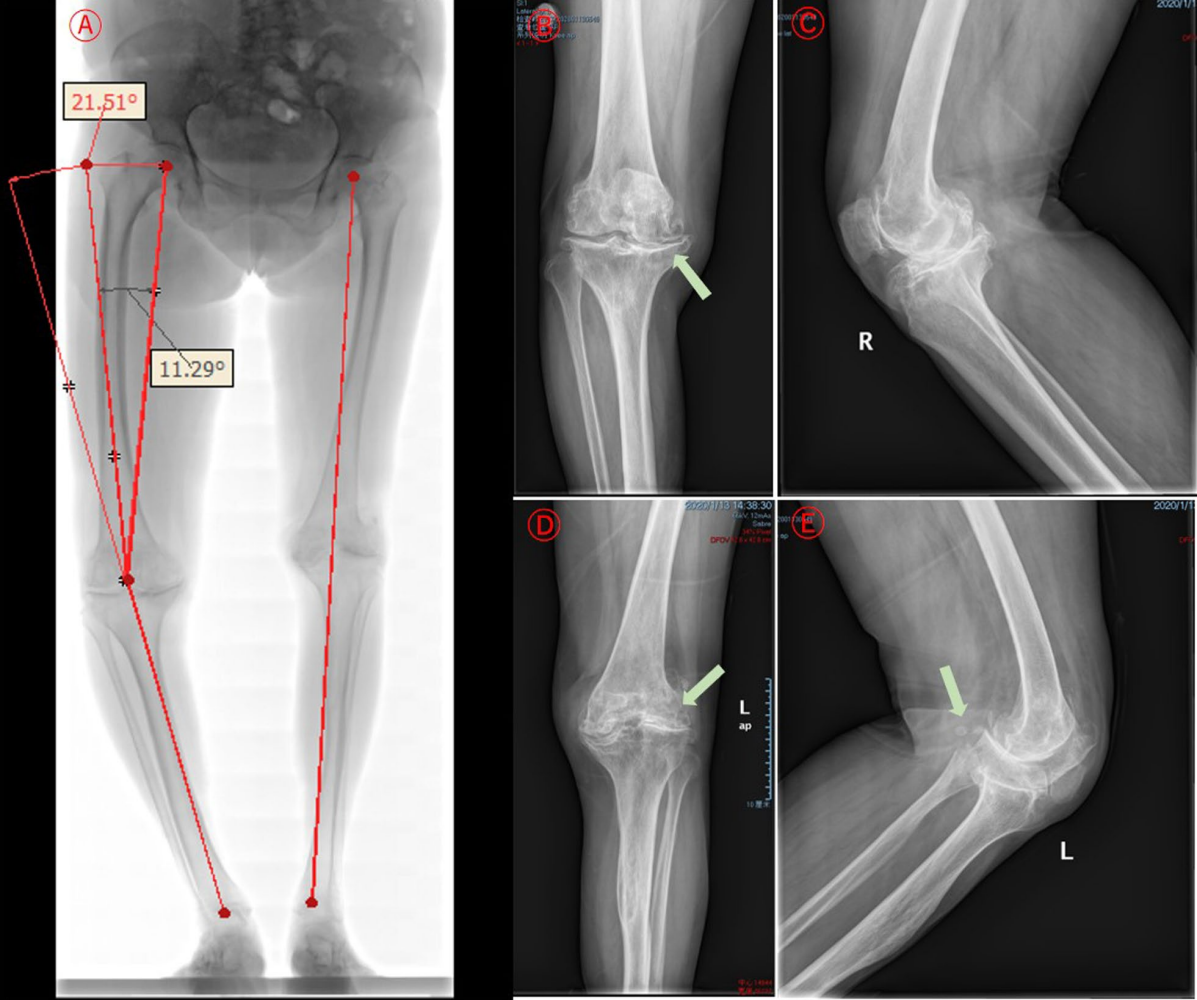
Characteristics of bilateral lower limb and knee DR at admission. **a** Long-leg radiograph showed bilateral femur and tibia dysplasia with deformation of the shafts. The right varus angulation was 21.5°, and the FMAA was 11.3°. The mechanical axis of the left lower limb was normal. **b**–**e** Bilateral knee joint AP & LAT showed that the extensive subchondral bones of the knees were sclerotic, and the articular surfaces were out of flatness. Ambilateral joint gaps were significantly narrowed, especially in the lateral left knee and medial right knee. A couple of loose bodies were seen in the left knee

X-ray examination revealed bilateral femoral condyles and proximal tibial dysplasia in this patient. Joint spaces of knees were narrowed, especially in the lateral left knee and medial right knee. The right varus angulation was 21.5°, and the femoral mechanical-anatomical angle (FMAA) was 11.3°. Bilateral knees with bone sclerosis were seen under the articular surfaces, joint surfaces were not smooth and had osteophytes, and a couple of loose bodies were seen in the left knee (Fig. 1).

This patient reported a contagious disease history of smallpox in childhood, and her bilateral knees became deformed after healing. The variola virus was eradicated in China in 1961, and pitted facial scars were visible on her face, which were typical of previous smallpox infection. The woman presented with progressive pain in the bilateral knees 8 years ago and underwent loose body removal for left knee secondary arthritis 7 years ago. Additionally, she underwent debridement and antituberculosis therapy for tuberculosis of the lumbar spine 40 years ago and recovered well without the occurrence of complications after the operation. Laboratory results showed that the white blood cell count (WBC), C-reactive protein (CRP), erythrocyte sedimentation rate (ESR), and procalcitonin (PCT) were normal. Lymphocyte percentage (44.0%), alkaline phosphatase (141.92 IU/L), γ-glutamyltransferase (95.45 IU/L), leucine aminopeptidase (46.62 IU/L), and glutamate dehydrogenase (10.47 IU/L) were slightly elevated, and the eosinophil count (0.03 × 10^9^/L) was slightly decreased.

Based on physical examination, imaging and past medical history, the patient was considered to have bilateral knee osteomyelitis variolosa combined with secondary arthritis. The patient underwent right total knee arthroplasty (TKA) owing to persistent pain with limited motion in the knees, and the right knee was more severe than the left knee. Postoperatively, she was given intravenous antibiotic therapy with piperacillin sodium and sulbactum sodium for prevention of infection. The woman recovered well, the surgical incision healed primarily, and the knee underwent regular functional exercises.

**Fig. 2:**
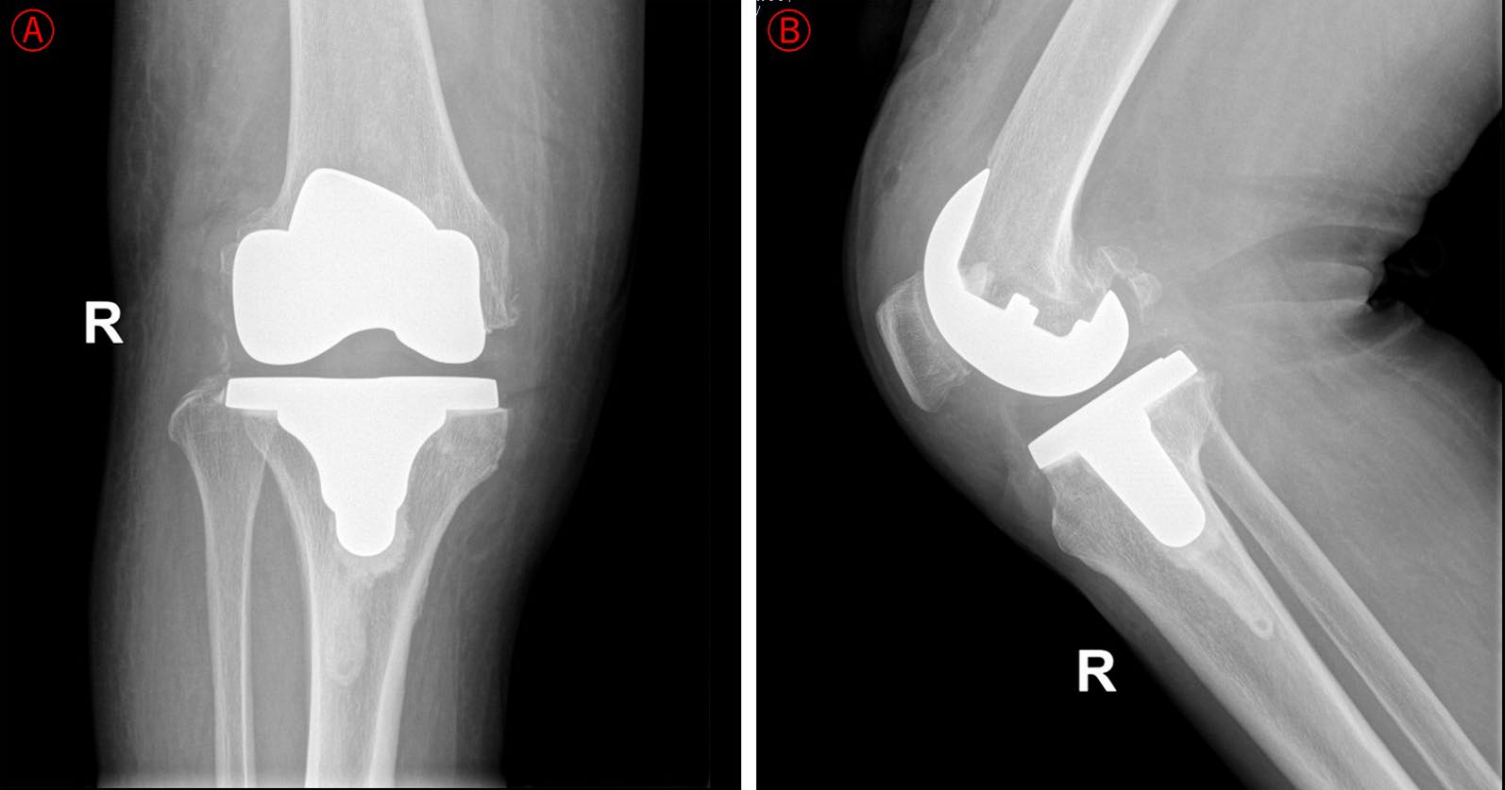
Results of the right knee DR at readmission. **a**–**b** Right knee joint AP & LAT showed that the surfacing prosthesis of the knee was in good position and stable

This elderly woman fell at home on the 20th postoperative day, resulting in a tear of the right knee proximal surgical incision. The patient was treated conservatively on her own for more than 1 month, but the wound continued to extravasate and did not heal. When she was readmitted to our hospital on the 52th day postoperatively, physical examination revealed a sinus tract in the proximal part of the right knee, with red inflamed skin and visible yellowish-white fluid exudation. X-ray examination showed a stable surfacing prosthesis of the knee (Fig. 2). Laboratory results showed that the WBC, CRP, ESR, PCT, and myeloperoxidase (MPO) levels were normal. The consequences of bacterial culture were methicillin-resistant coagulase-negative *Staphylococcus* (MRCNS). The bacteria were sensitive to vancomycin, gentamicin, and rifampicin and were resistant against clindamycin, levofloxacin, and penicillin.

The patient received extensive debridement under intravenous anesthesia on the third day of readmission. Fortunately, no evident infection or inflammatory tissue was seen in the articular cavity. The athroplasty was stable, the surrounding soft tissues were good, and there was no necrotic tissue. According to Tsukayama classification, the prosthetic joint infection was Type IIA. Therefore, we did not remove the knee prosthesis. Vancomycin anti-infective therapy was given postoperatively. Her surgical incision healing by second intention without recurrence of incision infection or sinus tract formation. At the 12-month follow-up, there was no recurrence of infection and no pain during movement. Under the correct guidance of the multidisciplinary rehabilitation programme, this elderly woman recovered good motor function of the right knee, and Hospital for Special Surgery (HSS) knee score was 88.

## Systematic review

### Methods

Data for the present study were identified through a PubMed (MEDLINE), Web of Science, and Google Scholar search of case reports published on osteoarticular sequelae of smallpox by two independent reviewers, using the subject heading “osteomyelitis variolosa”. We explored case reports published within a specific time period, starting and ending from 1980 (when the WHO declared smallpox eradicated) to 2021. Two reviewers screened titles and abstracts for selection of case reports; excluded all papers that were not clearly proven to be osteomyelitis variolosa by history, imaging, or laboratory examination; and eliminated all duplicate papers. We reviewed all case reports and extracted the following data from each paper: patient age, sex, major complications, therapeutic measures and outcome.

## Results

**Fig. 3:**
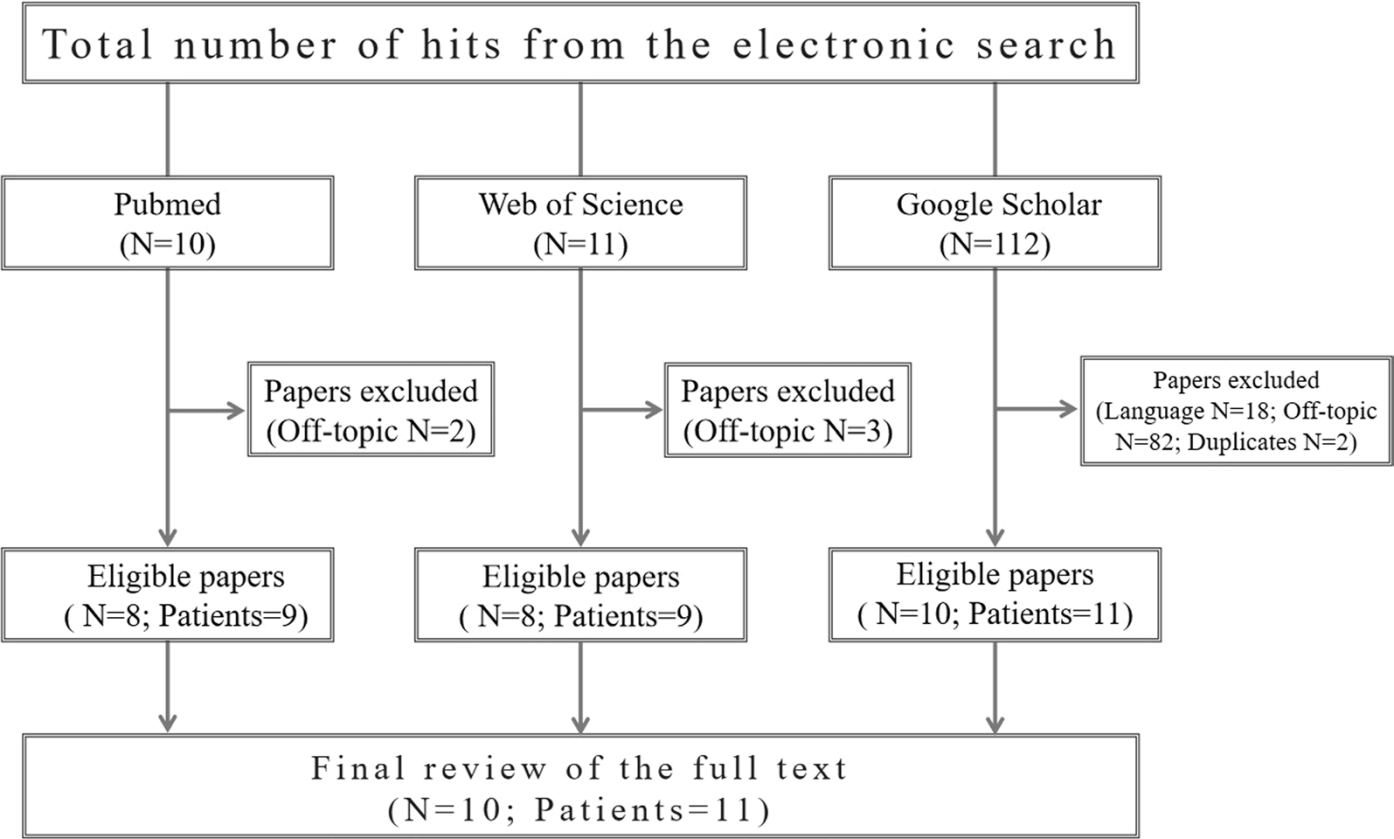
Flow chart depicting the systematic review process of the present study

In areas where smallpox was once endemic, there are still a certain number of patients with osteomyelitis variolosa. Clinicians often lack sufficient experience in the treatment of these diseases and are unable to give patients the correct treatment and effective means in a timely manner or even provide a misdiagnosis and mistreatment. After reviewing the literature, 10 papers and 11 finished cases were included (Fig. 3). Here, we present data from all cases of osteomyelitis variolosa [13–22]. We have combined the characteristics of the patients in the following paragraphs, and detailed data are available in the table below (Table 1).

**Table 1:** Clinical characteristics of cases of osteomyelitis variolosa

Case reference	Age	Sex	Major complications	Location	Management	Outcome
Arora [13]	64	Male	Closed fracture of the right humeral shaft	Elbows	Open reduction and bone graft with internal fixation	Bone healing
	53	Male	Elbow instability	Elbows	Splinting	Improvement
Andrews [14]	64	Male	Restriction of motion in elbows	Elbows	Not clear	Not clear
Balaji [15]	70	Female	Secondary arthritis of right knee	Elbows, ankles, right knee	Conservative management	Not clear
Purandarnath [16]	65	Female	Secondary arthritis of multiple joints	Elbows, ankles, wrists, distal radioulnar joints, subtalar joints	Conservative management	Not clear
Nema [17]	40	Male	Trimalleolar fracture with subluxation of the right ankle	Elbows, ankles	Not clear	Not clear
Singh [18]	41	Male	Fracture of the distal third of the left humerus with radio-carpal anatomical disruption	Elbows, ankles, wrists, distal radioulnar joints	Open reduction and bone graft with internal fixation	Bone healing
Balachandar [19]	54	Male	Right acute cubital tunnel syndrome	Elbows, ankles, distal radioulnar joints	Surgical excision of the ganglion cyst, release of the cubital tunnel retinaculum, and anterior transposition of the ulnar nerve	Improvement
Mugalur [20]	70	Male	Secondary arthritis of knees and ankles	Elbows, ankles, knees	Right ankle arthrodesis	Improvement
Thomas [21]	40	Male	Pain with activity in multiple joints	Elbows	Not clear	Not clear
Khurana [22]	56	Male	Asymptomatic elbow deformity	Elbows	Conservative management	Not clear
Present study	70	Female	Secondary arthritis of knees	Elbows, knees	Total knee arthroplasty, debridement and suturing	Improvement

## Discussion

### History

In 1873, Bidder first reported osteoarticular lesions of smallpox [23]. Nevertheless, the fact that smallpox affects bones has been recognized since at least 1568 [24]. Traces of osteoarticular sequelae of smallpox have been found on well-preserved bones excavated in a medieval cemetery [25]. Additionally, osteological evidence of osteomyelitis variolosa has been found in human remains from North America in the seventeenth century [24]. Chiari gave the name osteomyelitis variolosa to osteoarticular lesions of smallpox based on histological examination during the epidemic in Prague in the course of the winter of 1891–1892 [26], and this name has since been widely used by researchers. However, since the eradication of smallpox virus in 1980, osteomyelitis variolosa has rarely been reported by clinicians, and based on its specific imaging manifestations, which makes the management of this sequela difficult, our study only includes papers published after the eradication of variola virus (1980–2021).

### Joints

In the absence of a secondary infection, the end-stage periostitis during the progression of osteomyelitis variolosa either is organised and merged with the old or resorbed leaving a normal diaphyseal contour with some sclerosis [9]. Although the process of skeletal lesions caused by smallpox virus is irreversible [7], osteomyelitis variolosa is self-confining and is not life threatening. Skeletal deformity is often the outcome of this disease. Late manifestations of osteomyelitis variolosa are ankylosis, dislocation, subluxation, shortening and deformity of long and short tubular bones, flared metaphyses and precocious osteoarthritis [9]. However, the handicap of a single skeletal deformity on a patient’s motor function is relatively small. Based on this, we should focus our attention on the lesion site in the joint. It is known from studies during the smallpox epidemic that joint sites may vary widely but are usually symmetrical. Elbow deformities were present in at least 80% of patients, hands and wrists in 20%, and ankles and feet in 18%, while knees, hips and shoulders were less likely to be present [8], which is generally consistent with the present study. Our systematic review revealed that all patients presented with bilateral elbow deformities, the ankle was the second most common site of lesion at 50%, and knee lesions accounted for 25% of this study, but arthritis secondary to knee deformities was the overriding reason for patients to seek medical care.

### Imaging

The X-ray features of the elbow lesion in osteomyelitis variolosa were very diverse, mainly consisting of sclerosis of the peri-elbow bone, long slender flexuous shaft of the humerus, ulna and radial tuberosity, and dysplasia or enlargement of the distal humerus, olecranon and radial head. Alternatively, the articular space of the elbow is reduced, the joint is misshaped or dislocated, and the proximal radius and ulna are fused [13–20, 22]. The elongated medial and lateral condyles of the humerus are visible in some patients [13], and a significantly lengthened humeral ectocondyle has also been seen in unearthed human medieval remains [25]. Imaging findings of the ankle show deformities of the distal tibia and fibula, as well as collapse and resorption of the talus, reduction of the articular space, and ankylosis with arthritis [15–17, 20]. The X-ray appearances of the knee lesion are dysplasia of the femoral condyle, narrowing of the joint gap, and arthrophlogosis details such as subchondral bone remodeling, hyperostosis, and osteophyte formation [15, 20]. Imaging of our case showed bilateral femoral condyles and proximal tibial dysplasia with deformation of the shaft of the femur and tibia. The extensive subchondral bones of both knees were sclerotic, and the articular surfaces were were out of flatness. The right varus angulation was 21.5°, and the FMAA was 11.3°. The joint gap was significantly narrowed, with the medial side being the most remarkable. The mechanical axis of the left lower limb was normal, but the lateral joint space was narrowed.

### Complications

For elbow sequelae triggered by osteomyelitis variolosa, flexion deformities and activity limitations are the most frequent problems [14, 16, 17, 19–22]. Although some patients have exaggerated clinical deformities and imaging abnormalities of the elbow, joint function is not obviously limited, and they are still able to perform manual labor and lift heavy weights using both upper limbs [20, 22]. A patient with elbow flexion deformities developed right acute cubital tunnel syndrome and was treated with surgical excision of the ganglion, release of the cubital tunnel retinaculum, and anterior transposition of the ulnar nerve, which resulted in the effective improvement of ulnar nerve function [19]. Elbow instability is the next most common elbow complication, and its primary cause is attributed to hypoplasia of the distal humerus and elongation of the medial and lateral condyles [13, 15, 18]. Secondary osteoarthritis due to articular malformation is a central cause for seeking medical care for patients. One patient was misdiagnosed with rheumatoid arthritis (RA) due to secondary arthritis of multiple joints and was treated conservatively after the diagnosis was clarified [16]. Another patient underwent ankle arthrodesis for secondary arthritis of the right ankle and recovered well after surgery [20]. A total of three cases, including the present study’s case, sought doctors’ office visits for secondary arthritis of the knee [15, 20], which has a great impact on quality of life. Our patient received TKA, and the other two patients opted for conservative treatment. However, based on medical history and imaging, the knee lesions in two patients did not rule out the possibility of primary knee osteoarthritis [15, 20]. In addition, two patients suffered humeral fracture, and one patient suffered a trimalleolar fracture with subluxation of the right ankle [13, 17, 18]. Osteomyelitis variolosa can cause substantial changes in the mechanical properties of cortical bone, trabecular bone, and the entire skeleton [27]. An abnormal mechanical axis and juxta-deformity stress rise at the bone may be responsible for the susceptibility to fracture [18]. Despite distortion of the medullary structure, two patients with humeral fractures healed well with repositioned internal fixation.

### Viral arthritis

During the global pandemic of an emerging infectious disease, namely, coronavirus disease 2019 (COVID-19), caused by severe acute respiratory syndrome coronavirus 2 (SARS-CoV-2) [28], researchers have gained new insights into motor system diseases due to viruses [29]. Numerous viruses have been implicated as the causative agent of viral arthritis, including parvovirus B19, hepatitis A (HAV), B (HBV) and C virus (HCV), rubella virus, alphaviruses, flaviviruses, and retroviruses [30]. Viral arthritis is usually self-limiting and does not require specific intervention. Although symptoms can be prolonged in rare cases [31], this feature is consistent with the self-limitation of osteomyelitis variolosa. However, it is worth noting that the joint manifestations of osteomyelitis variolosa differ in that it is destructive, unpreventable and untreatable [7].

### Pathogenesis

The mechanisms by which viruses cause osteoarticular diseases are diverse and still poorly understood but are clearly influenced by both host and viral factors [32]. For example, the systemic inflammatory response may exert a significant part in the histophysiology of the bones and joints in COVID-19 patients [29]. Although the major musculoskeletal complications of severe acute respiratory syndrome (SARS) are osteonecrosis and reduced bone mass, these are not caused by the disease itself. These sequelae are caused by side effects following steroid pulse treatment for SARS [33]. Nevertheless, we have some evidence that the pathological changes of osteomyelitis variolosa are caused by the smallpox virus. The reasons for attributing bone infections to variola viruses have been fully considered by Cockshott, MacGregor and Davidson and may be briefly summarized as follows: (1) the lesions behave like proven virus infections of bone; (2) elementary bodies are present in the fluid from affected joints, which is usually sterile on culture for pyogenic organisms; (3) the clinical course is quite different from that of acute bacterial osteomyelitis; (4) antibiotics and chemotherapy fail to prevent the development of these bone changes or to influence their course; and (5) the radiological distribution and appearance of bone infection is unlike that of simple osteomyelitis and is characteristic of smallpox [7, 34]. Unfortunately, however, a detailed histopathological description of osteomyelitis variolosa is lacking [35].

### Antidiastole

Osteomyelitis variolosa is a self-limiting illness that cannot be prevented or repaired [7], so the diagnosis of this disease is crucial. RA and other types of systemic inflammatory arthritis may be difficult to distinguish from viral arthritis, but these diseases require early intervention to improve long-term prognosis [31]. Purandarnath reported a case of osteomyelitis variolosa misdiagnosed as RA [16]. This patient had bilateral wrist, elbow, and ankle pain and stiffness and was positive for rheumatoid factor (RF). However, the patient’s imaging was not entirely consistent with RA and was negative for anti-cyclic citrullinated peptide (CCP). Because of the lack of etiological evidence, the diagnosis of osteomyelitis variolosa currently needs to be considered from multiple aspects, combining X-ray images [9], clinical manifestations, careful physical examination, history of smallpox and laboratory inspections relevant to the differential diagnosis [10]. Imaging manifestations of osteomyelitis variolosa are mainly shortening of the long bones and short bones, flail joints, subluxations, dislocations, gross deformities of bones, precocious osteoarthritis and ankylosis [9]. Nevertheless, variola virus has been eradicated for more than 40 years [1], and osteomyelitis variolosa has become a challenging diagnostic problem. Physicians and patients do not always associate it with past contagious disease history, which often leads to missed diagnosis of this disease. Various illnesses, such as achondroplasia, pseudohypoparathyroidism, and Caffey disease, have similar clinical features and imaging manifestations to osteomyelitis variolosa (Table 2). Inquiry regarding a history of smallpox is recommended when unusual joint deformities and growth inequalities of the extremities are present [10].

**Table 2:** Differential diagnosis for osteomyelitis variolosa

Disease	Age tendency	Sex predilection	Etiology	Unilateral or Bilateral	Imaging	References
Osteomyelitis variolosa	Children	No sex differences	Variola virus	Bilateral	Ankylosis; dislocation; subluxation; shortening and deformity of long and short tubular bones; flared metaphyses and precocious osteoarthritis	[8, 9]
Achondroplasia	Infants	No sex differences	Genetic factors	Bilateral	Short, robust tubular bones; squared off iliac wings; flat, horizontal acetabula; marked narrowing of the sacrosciatic notch; a characteristic proximal femoral radiolucency; narrowing of the interpediculate distance of the caudal spine; short proximal and middle phalanges	[36]
Pseudohypoparathyroidism	Children and adults	No sex differences	Genetic factors	Bilateral	Ectopic ossifications; shortening of the metacarpals and metatarsals	[37, 38]
Sequelae of septic arthritis	Children	Male	Bacterial infection	Unilateral	Difference in limb size; avascular necrosis of the femoral head; pathological fracture	[39]
Osteoarthritis	Middle-aged and older	Female	Degeneration	Bilateral	Narrowing of the joint space width; osteophyte formation; development of subchondral sclerosis and cysts	[40]
Rheumatoid arthritis	Any age	Female	Chronic inflammatory disease	Bilateral	Joint space narrowing; bone erosion; subluxation; ankylosis; mutilating changes	[41]
Caffey disease	Infant	No sex differences	Genetic factors	Bilateral	Periosteal new bone formation leads to cortical thickening (hyperostosis) of the affected bones and swelling of the overlying soft tissue	[42]
Leprosy	Adults	No sex differences	Mycobacterium leprae	Bilateral	Juxta-articular erosions; periostitis; bone resorption; sacroiliitis; deformed joints	[43, 44]
Tuberculosis	Children and the elderly	No sex differences	Mycobacterium tuberculosis	Unilateral	Joint space narrowing; juxta-articular osteoporosis; peripherally located osseous erosions	[45]
Kashin-Beck disease	Children	No sex differences	Selenium deficiency and cereal contamination	Bilateral	Symmetrical enlargement of the phalanges; brachydactyly; joint deformity and even dwarfism	[46, 47]

### Treatment

The treatment options for the skeletal sequelae of patients with osteomyelitis variolosa are based on a combination of symptoms, disease progression, and the patient’s demand for quality of life. Elbow deformity combined with flexion contracture or instability is the most frequent sequelae of osteomyelitis variolosa [8]. For patients whose elbow function is not significantly affected and who do not yet have severe pain [20, 21], conservative treatment is probably the best option. Total elbow arthroplasty (TEA) is an effective treatment choice for patients with severe pain or instability of the elbow or for patients requiring high-quality motion [48]. However, there is a risk of long-term complications after TEA, such as aseptic loosening, infection, and polyethylene or bushing wear [49]. Elbow deformity in patients with osteomyelitis variolosa is often combined with distal humeral dysplasia, lengthening of the condyles of the humerus, and distortion of the long bones [13, 25], which further increases the operating difficulty of TEA. For the pain associated with knee secondary arthritis and the limitation of motion, it becomes the leading reason to seek medical advice for patients with osteomyelitis variolosa in the presence of knee deformity [15, 20]. In young and active sufferers with osteoarthritis of moderate radiographic severity, knee osteotomy can be used, delaying TKA by 10 years in more than 85% of patients [50]. TKA is one of the most commonly performed and cost-effective musculoskeletal surgical procedures for end-stage patients with nasty ache, severe abnormality, and significant narrowing of the joint space on imaging [51] and can significantly improve physical function and long-term pain alleviation [52]. Our patient is the first reported case of knee secondary arthritis of osteomyelitis variolosa treated with TKA. The patient recovered well in the early postoperative period, with improvement in knee varus and arthralgia. However, similar to TEA, there is a risk of postoperative complications such as aseptic loosening, deep infection, periprosthetic fracture, and PE wear [53]. Our patient later developed knee infection due to trauma. Fortunately, the infection was cured after radical debridement. In the case of osteomyelitis variolosa combined with fracture, although the medullary structure of the long bone is deformed, good fracture healing can still be achieved after open reduction and internal fixation [13, 18].

## Conclusion

In conclusion, osteomyelitis variolosa differs from ordinary viral arthritis in that the skeletal complications it causes are destructive, unpreventable and untreatable. Although this disease is not a direct threat to the safety of patients, severe skeletal deformities can have a significant impact on quality of life. As society progresses and living standards continue to improve, people are demanding higher quality motor function. With advances in surgical techniques, clinicians are offering an increasing number of treatment options for patients with osteomyelitis variolosa. However, most importantly, smallpox has basically been removed from the historical arena, and it is difficult for us to make an accurate diagnosis of osteomyelitis variolosa in the first place. The frog in the well knows nothing of the great ocean, so clinicians need to continuously improve their clinical cognition of this disease and further sum up their experience in diagnosis and treatment.

## Data Availability

All data are available through cited literature.

## Abbreviations

WHO: World Health Organization
MMT: Manual muscle testing
FMAA: Femoral mechanical-anatomical angle
WBC: White blood cell count
CRP: C-reactive protein
ESR: Erythrocyte sedimentation rate
PCT: Procalcitonin
TKA: Total knee arthroplasty
TEA: Total elbow arthroplasty
MPO: Myeloperoxidase
MRCNS: Methicillin-resistant coagulase negative *Staphylococcus*
HSS: Hospital for special surgery
DR: Digital radiography
RA: Rheumatoid arthritis
COVID-19: Coronavirus disease 2019
SARS: Severe acute respiratory syndrome
SARS-CoV-2: Severe acute respiratory syndrome coronavirus 2
HAV: Hepatitis A virus
HBV: Hepatitis B virus
HCV: Hepatitis C virus
RF: Rheumatoid factor
CCP: Anti-cyclic citrullinated peptide
